# Distribution Pattern and Change Prediction of *Luprops orientalis* (Coleoptera: Tenebrionidae) Suitable Area in East Asia Under Climate Change

**DOI:** 10.3390/insects16060626

**Published:** 2025-06-13

**Authors:** Jieqiong Wang, Shuangyi Wang, Yunchun Li, Shuangmei Ding, Zhonghua Wei, Aimin Shi, Ding Yang

**Affiliations:** 1 The Key Laboratory of Southwest China Wildlife Resources Conservation of the Ministry of Education, College of Life Sciences, China West Normal University, Nanchong 637009, China; jqwang0902@126.com (J.W.); liyc2260@cwnu.edu.cn (Y.L.); aiminshi2003@126.com (A.S.); 2 Student Affairs Department, Sichuan University of Arts and Science, Dazhou 635000, China; wsy_ws1@163.com; 3 The Institute of Scientific and Technical Research on Archives, National Archives Administration of China, Beijing 100050, China; shuangmeiding@163.com; 4 State Key Laboratory of Green Pesticides, Guizhou University, Guiyang 550025, China; 5 State Key Laboratory of Agricultural and Forestry Biosecurity, MARA Key Lab of Surveillance and Management for Plant Quarantine Pests, College of Plant Protection, China Agricultural University, Beijing 100193, China

**Keywords:** climate change, *Luprops orientalis*, MaxEnt, pest, suitable area

## Abstract

*Luprops orientalis* (Motschulsky, 1868) is an economically important pest in traditional Chinese medicines, widely distributed in East Asia. However, the primary limiting factors affecting its distribution, potential suitable areas, as well as its response to global warming, remain largely unknown. Utilizing 295 filtered distribution points and 10 environmental variables (9 climate variables and 1 land cover type), this study uses the MaxEnt model to predict the potential distribution of *L. orientalis* under near-current and future environmental change scenarios. The results indicated that precipitation of the warmest quarter (bio18), temperature seasonality (bio04), and precipitation of the wettest month (bio13) were the most significant environmental variables affecting the distribution of suitable habitats for *L. orientalis*, while the contribution of average variation in daytime temperature (bio2) was the smallest. Under the near-current climate, the areas of low, moderate, and high suitability for *L. orientalis* are approximately 1.02 × 10^6^ km^2^, 1.65 × 10^6^ km^2^, and 8.22 × 10^5^ km^2^, respectively. The suitable areas are primarily located in North China, Central China, the Korean Peninsula, and Central and Southern Japan. Under future climate conditions, the potential suitable areas are expected to expand significantly, especially in Central China. However, the high-suitability areas in North China are predicted to experience a slight reduction. With the increase in carbon emission concentrations, the suitable area shows an increasing trend in the 2050s, followed by a declining trend in the 2090s. The centroids of suitable areas will shift to the northeast in the future. These findings enhance our understanding of how climate change affects the distribution of *L. orientalis* and will assist governments in formulating effective pest control strategies, including widespread monitoring and stringent quarantine measures.

## Simple Summary

*Luprops orientalis* (Motschulsky, 1868) is an important pest in traditional Chinese medicines and can cause significant economic losses and degrade its quality. In this study, the maximum entropy (MaxEnt) model was employed to predict the distribution of *L. orientalis* under both near-current and future environmental scenarios in East Asia. Under near-current environmental conditions, the suitable areas for *L. orientalis* are primarily distributed in North China, Central China, the Korean Peninsula, and Central and Southern Japan. In China, high-suitability areas are mainly distributed in Hebei, Liaoning, Jiangxi, Shaanxi, and Hunan. Under future environmental scenarios, the centroids of suitable areas are expected to shift towards higher latitudes, with the overall suitable area increasing, while the highly suitable areas decrease. Additionally, as carbon emission concentrations rise, the suitable areas are expected to diminish in the 2090s.

## 1. Introduction

Global warming is an indisputable fact and a major scientific issue of this century. Climate change is expected to alter the geographic distribution patterns of many species, which in turn will affect the structure and function of ecosystems [1]. Studies have shown that global climate change is contributing to the escalating loss of biodiversity [2] and altering the distribution ranges of various species [3]. Therefore, investigating the potential geographic distribution of species under climate change provides a theoretical basis for biodiversity conservation and resource management. Furthermore, it serves as an essential tool for predicting the impact of climate change on species distribution patterns.

Species distribution models (SDMs) primarily utilize known species distribution points and environmental variables to estimate the ecological niche of species based on specific computational methods. Subsequently, these models project this niche onto the landscape to predict the probability of species occurrence in target areas [4,5]. Commonly used SDMs include the maximum entropy (MaxEnt) model, Bioclimate Analysis and Prediction System (BIOCLIM), Ecological Niche Factor Analysis (ENFA), Genetic Algorithm for Rule-set Production (GARP), Generalized Linear Model (GLM), and Generalized Additive Model (GAM) [6,7,8,9,10]. Among these, the MaxEnt model is the most widely used for suitability analysis. It can quantitatively predict geographic distribution by leveraging limited geographic distribution points and climate environmental factors, even when the correlation between these two variables is unclear [11]. The prediction results partially depict the fundamental ecological niche of species, which can then be projected onto a geographic area to identify the potential suitable locations for those species.

The Tenebrionidae is one of the largest families in Coleoptera, comprising approximately 30,000 species across 11 subfamilies worldwide [12,13]. The feeding habits of tenebrionid species encompass herbivory, carnivory, omnivory, and detritivory, among others. Some species directly or indirectly damage stored products, making them closely related to human economic interests. Within this family, 227 species are identified as storage pests [14,15], constituting a significant component of stored-product pests. Among them, *Luprops orientalis* (Motschulsky, 1868) is considered as an economically important pest in traditional Chinese medicines [14], frequently appearing in pest lists [15,16,17]. It is a flight-capable beetle with a strong dispersal capacity, widely distributed in East Asia. *Lyprops sinensis* Marseul, 1876 is commonly included in the lists of stored-product pests in China; however, it is a synonym of *Luprops orientalis* [15,18,19]. Field surveys indicate that both the larvae and adults of this species feed on plant detritus in the natural environment, with many populations occurring near villages. Currently, its potential suitable area and response to climate change are unknown.

Accurate prediction of the potential suitable area for this pest not only aids preventing further spread but also effectively reduces management costs [20,21]. In this study, the MaxEnt model was used to predict the potential suitable areas of *L. orientalis* under climate change scenarios in East Asia. The aims of this study are to identify the dominant environmental factors affecting the geographical distribution of *L. orientalis* and to assess the impact of climate change on the suitable habitat areas for this species.

## 2. Materials and Methods

### 2.1. Species Occurrence Data

The distribution points were collected from field investigations and species occurrence databases. From May 2016 to June 2020, field investigations were conducted in North China, and 64 distribution points were recorded [19]. An additional 313 and 12 occurrence sites of *Luprops orientalis* were obtained from the Global Biodiversity Information Facility (GBIF, https://www.gbif.org) and the China National Knowledge Infrastructure (https://www.cnki.net/), respectively. A total of 389 distribution points were obtained. This methodological design specifically targets the mitigation of both autocorrelation effects and the accumulation of sampling bias—two critical factors that contribute to predictive model overfitting [22]. To avoid overfitting caused by sampling deviation, we conducted secondary sampling of points on a 2.5-arc grid to reduce sampling deviation and minimize the possible impact of spatial autocorrelation [22]. Ultimately, 295 occurrence records of *L. orientalis* were selected to construct the predictive model.

### 2.2. Environmental Variables

The near-current and future bioclimatic variables were downloaded from WorldClim (Version 2.1, http://www.worldclim.org/) at a spatial resolution of 2.5 arcminutes.

**Figure 1:**
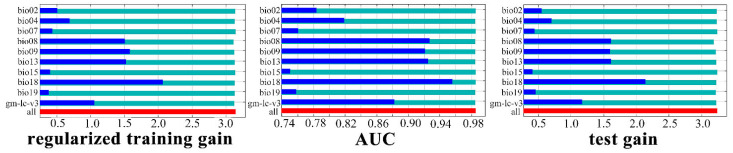
Jackknife test used for evaluating the importance of environmental variables.

The near-current climate data we used included 19 bioenvironmental variables measured between 1970 and 2000. And a total of 20 environmental variables (19 worldclim bioclimatic variables, and 1 land cover type that can be downloaded from https://globalmaps.github.io/glcnmo.html, accessed on 12 April 2025) were used for current and future model predictions. The date type, resolution, and data source of the land cover version 3 are Byte (8 bits), 15 arcseconds, and MODIS data 2013 (Terra & Aqua), respectively [23]. The collinearity of bioclimatic variables can affect the accuracy of the MaxEnt model, contributing to an inappropriate extent of simulated species distribution [24,25,26]. The jackknife method was used to assess the contribution rates of the environmental variables to the predictive model, and variables with a contribution rate of zero were subsequently removed. Through systematic screening, 10 environmental variables (comprising 9 bioclimatic variables and 1 land cover type) were retained for the MaxEnt modeling analysis (Figure 1).

Future bioclimatic variables were provided by the Coupled Model Intercomparison Project Phase 6 (CMIP6) for the periods of 2041–2060. For the emissions scenarios, SSP1-2.6, SSP2-4.5, and SSP5-8.5 were used, representing low, moderate, and high greenhouse gas emission scenarios, respectively, for future climate projections. The Beijing Climate Center Climate System Model version 2 (BCC-CSM2-MR) was chosen as the future climate model [27], due to its effective simulation of extreme temperature indices and trends in China [28].

### 2.3. Modeling Methods

The world map used in this study was obtained from the Resource and Environment Science and Data Center (https://www.resdc.cn/, accessed on 18 March 2025). The MaxEnt v3.4.1 was employed to predict suitable areas for *Luprops orientalis* in East Asia. To reduce multicollinearity among variables, we used ArcGIS version 10.2 software and SPSS version 20.0 software to process and analyze the environmental variables, and used Pearson’s correlation test (Pearson’s r) for factor screening. The Pearson correlation coefficients (Pearson’s r) of the cross-correlations among the 20 variables were calculated using the SPSS version 20.0 software. In choosing one of them, when |r| > 0.85, it indicates that there is a collinear relationship. The prediction results of the Maxent model are significantly correlated with the parameter settings. The results obtained by using the default parameter settings may deviate greatly from the actual situation. Therefore, it is necessary to optimize the parameter settings according to the actual situation of the species [29]. The number of model iterations was set to 500 times, with 10,000 background points. In total, 75% of the species distribution points were randomly selected for model construction, while the remaining 25% were set aside for model testing. The result obtained after running the model is the average of the 10 test results and then the jackknife test was used to assess the importance of the variables [30]. Judging from the operation results, the area under the curve (AUC) values are all at a relatively high level, and the credibility is relatively high.

The ROC curve is used to evaluate the predictive performance of the model. The AUC is not affected by the threshold value and is commonly used to assess the accuracy of predictive models [31]. The AUC value ranges from 0 to 1, where an AUC < 0.5 indicates model failure, 0.5 < AUC < 0.7 indicates poor model accuracy, 0.7 < AUC < 0.8 indicates moderate accuracy, 0.8 < AUC < 0.9 suggests high accuracy, and AUC ≥ 0.9 denotes excellent accuracy [32,33]. In ArcGIS, the Jenks natural breaks classification method was used to classify and visualize the suitable areas for *Luprops orientalis*. The suitable areas were divided into four categories: *p* < 0.2 as unsuitable areas, 0.2 ≤ *p* < 0.4 as low-suitability areas, 0.4 ≤ *p* < 0.6 as moderate-suitability areas, and 0.6 ≤ *p* < 1 as high-suitability areas. The suitable areas were quantified using SDM Toolbox version 2.4.

## 3. Results

### 3.1. Model Optimization and Accuracy Evaluation

Based on the filtered dataset of 295 occurrence records and 10 environmental factors (Table 1), the results of the MaxEnt model demonstrated that the AUC value for the near-current period (1970–2000) reached 0.98 (Figure S1). The AUC value exceeding 0.9 showed that the MaxEnt model had excellent predictive performance, effectively highlighting the potential distribution range of *Luprops orientalis* under near-current and future climate conditions, along with its changing trends.

### 3.2. Dominant Environmental Variables and Their Response Curves

The importance of the environmental variables was evaluated using the jackknife method in Maxent (Figure 1). The results showed that among the 10 environmental variables, the top 3 contributors were bio18, bio04, and bio13, with contribution rates of 61.30%, 22.20%, and 9.00%, respectively (Table 1). Conversely, the contribution rate of bio02 was the lowest, at 0.2%, while the contribution rate of the land cover type was 0.8%. According to the permutation importance rates, bio13, bio09, and bio08 emerged as the dominant environmental variables, with rates of 43.60%, 17.50%, and 14.20%, respectively. Thus, based on the contribution and permutation importance rates, bio18 was identified as the most important environmental variable, whereas bio15 was determined to be the least important environmental variable.

**Figure 2:**
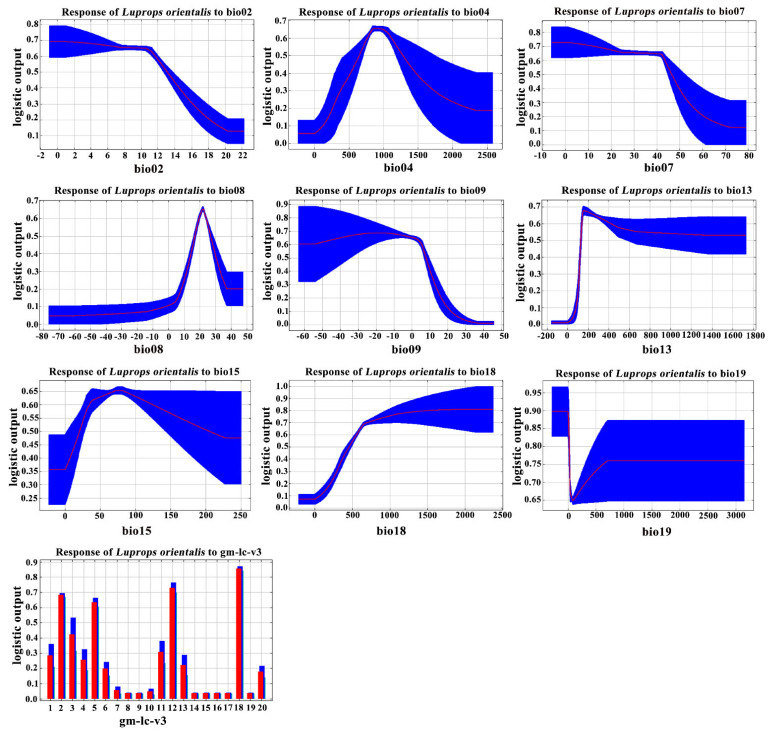
Response curves between the existence probability of *Luprops orientalis* and variables.

Response curve analyses of the selected bioclimatic variables—bio18, bio04, and bio13—were performed to explore their relationship with the distribution of *Luprops orientalis* (Figure 2). The presence probability of *L. orientalis* exceeds 0.5 when bio18 ranges from 565.73 mm to 886.55 mm, with the highest presence probability of 0.72 occurring at a precipitation of 726.14 mm. For bio04, the presence probability exceeds 0.5 within the range of 821.63 to 1068.67, with the peak presence probability of 0.65 observed at 1018.15. For bio13, the presence probability is greater than 0.5 when it ranges from 219.93 mm to 355.67 mm, with the highest presence probability of 0.62 occurring at 310.43 mm.

### 3.3. Near-Current Suitable Areas for Luprops orientalis

**Figure 3:**
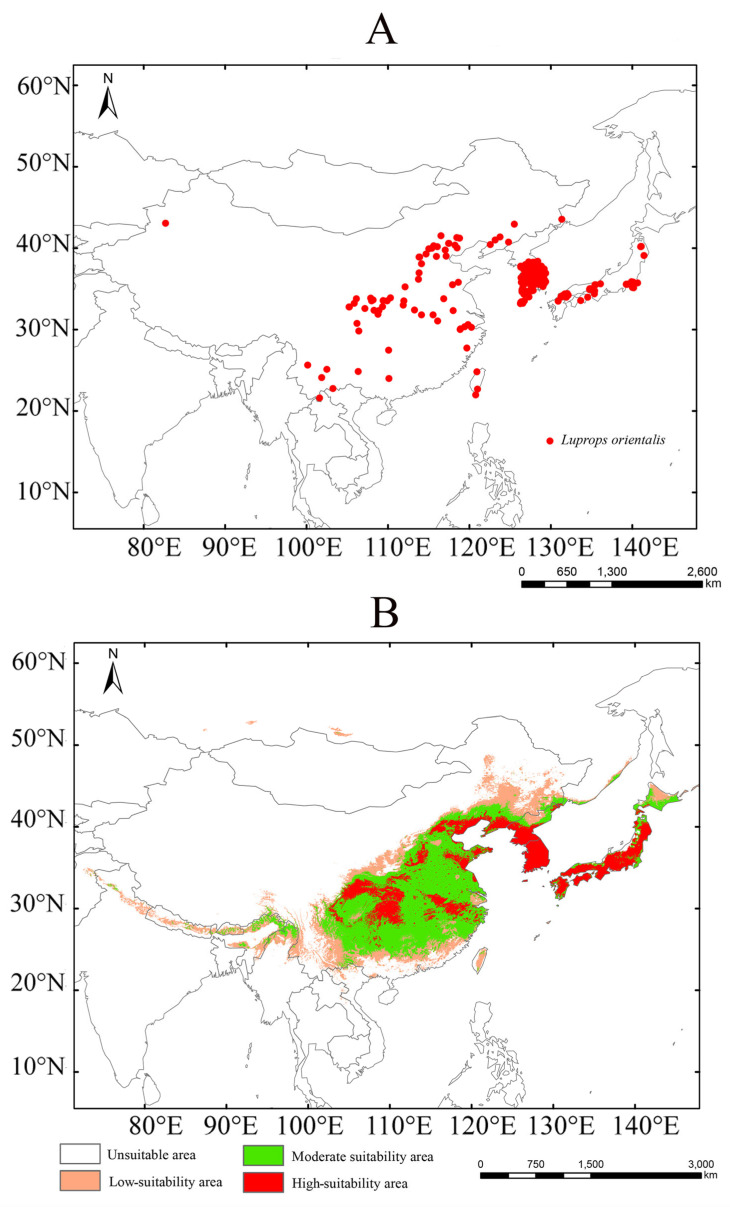
The occurrence records of Luprops orientalis (**A**) and suitable habitats for *L. orientalis* under the near-current climate (**B**).

Under near-current climatic conditions, the suitable areas for *Luprops orientalis* are classified into low-, moderate-, and high-suitability categories, encompassing approximately 1.02 × 10^6^ km^2^, 1.65 × 10^6^ km^2^, and 8.22 × 10^5^ km^2^, respectively (Table S2). The high-suitability areas are primarily concentrated in North China, Central China, the Korean Peninsula, and the Central and Southern regions of Japan (Figure 3). Low-suitability areas are mainly found along the southern slopes of the Himalayas, and in the peripheral regions of North China, Central China, the southern part of Northeast China, and Northern Japan. Notably, there are no high-suitability areas in Northwest China, Southwest China, Mongolia, or Russia, indicating a minimal invasion risk in these regions.

### 3.4. Changes in Suitable Areas for Luprops orientalis Under Future Climate Conditions

**Figure 4:**
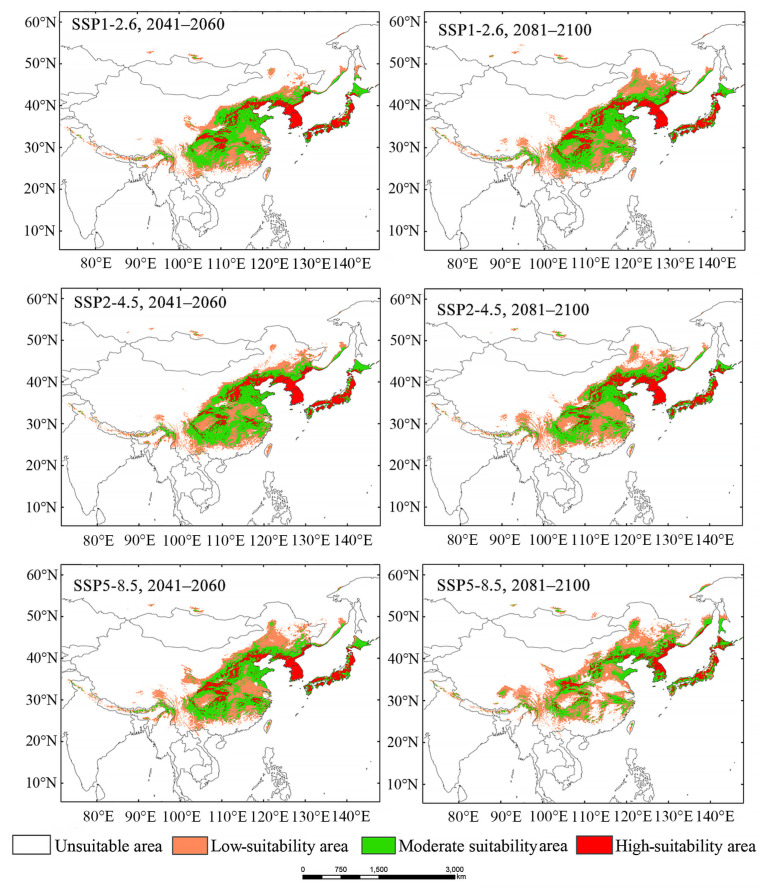
Suitable areas for *Luprops* orientalis under SSP1-2.6, SSP2-4.5, and SSP5-8.5 in 2041–2060 and 2081–2100, respectively.

Under future climate conditions, suitable areas for *Luprops orientalis* will predominantly be distributed across Central China, Northern China, Northeastern China, the Korean Peninsula, and the Japanese archipelago (Figure 4). Compared to its near-current distribution, the total suitable areas for *L. orientalis* will significantly increase (Table S2). In the 2050s, the suitable area will expand by 1.47%, 1.55%, and 2.70%, under the three carbon emission scenarios (SSP1-2.6, SSP2-4.5, and SSP5-8.5, respectively). In the 2090s, these increases are projected to be 2.47%, 1.29%, and 1.93%, respectively. Future climate conditions will result in a significant increase in low-suitability areas with rising carbon emission concentrations, while the moderate- and high-suitability areas will slightly decrease (Figure 4).

**Figure 5:**
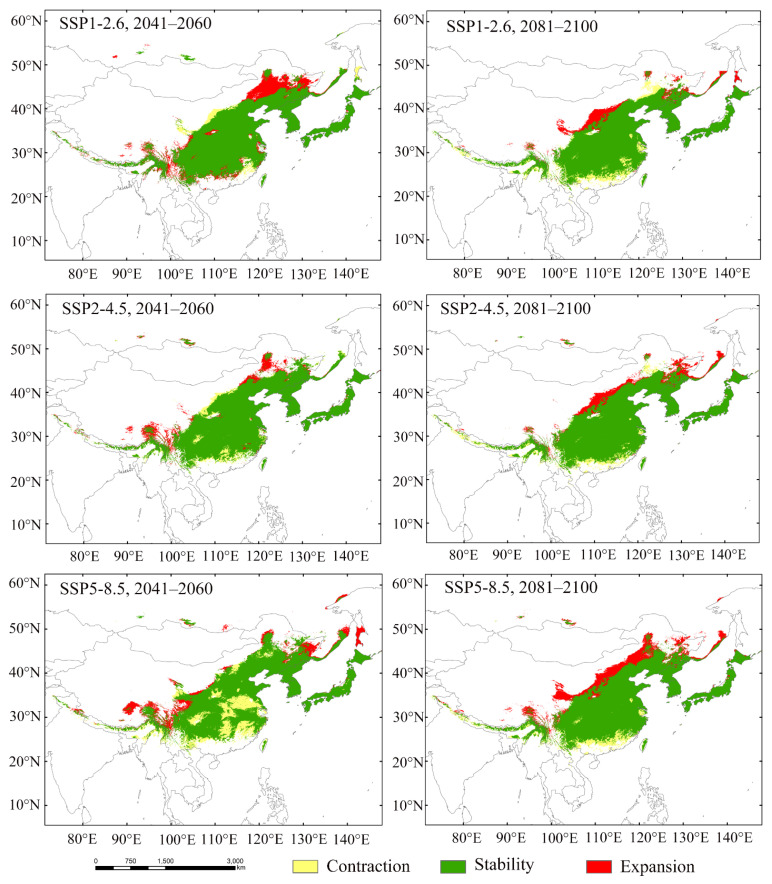
Suitable area changes for *Luprops orientalis* during 2041–2060 and 2081–2100.

The simulation results of the binarized MaxEnt model were applied to analyze spatial pattern changes in suitable habitats. The expansion of suitable areas for *Luprops orientalis* is mainly observed in Northeast China and Southwest China (Figure 5). A few suitable areas were also found on the southern slope of the Himalayas. The low-suitable area in Northeast China is the primary region where the suitable habitats for *L. orientalis* are expected to increase. Under the SSP1-2.6 scenario for the 2050s, the suitable area in Northeast China is significantly increased. In the case of SSP2-4.5, expansion will primarily occur in Northeast China and North China during the 2050s, while in the 2090s, it will extend further into Northeast China and the Hengduan Mountains region. Under the SSP5-8.5 scenario, a suitable habitat expansion area is anticipated mainly in North China during the 2050s and in the Hengduan Mountains, Northeast China, and southeastern Russia by the 2090s. The distribution pattern of *L. orientalis* underscores the substantial influence of carbon emissions on its suitable areas. Specifically, under SSP1-2.6 and SSP2-4.5, the suitable habitats for *L. orientalis* significantly increase in the 2050s. However, for SSP5-8.5, the total suitable area in the 2050s is markedly smaller than that in the 2090s.

### 3.5. Shift in the Centroids Within the Suitable Area

**Figure 6:**
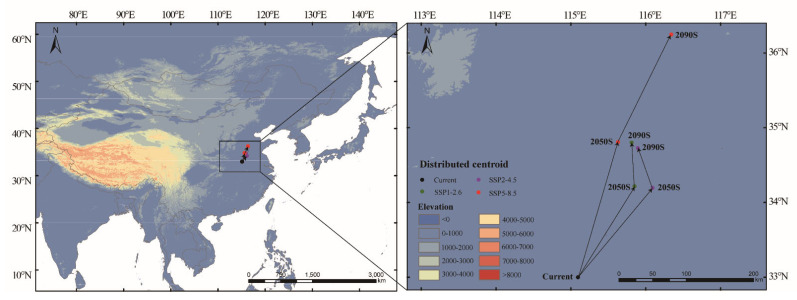
Shift in geometric center in suitable area under future climate scenarios.

Currently, the distribution center of the suitable area for *Luprops orientalis* is located in Fuyang City, Anhui Province, China (115.09479°E, 32.99922°N). Under the SSP1-2.6 and SSP2-4.5 scenarios, the geometric center first migrates northeast in the 2050s and subsequently shifts northwest in the 2090s (Table S3). In contrast, under the SSP5-8.5 scenario, the geometric center migrates northeast during both the 2050s and 2090s (Table S3). Overall, the centroids shift a relatively short distance, generally maintaining a movement towards the northeast (Figure 6).

## 4. Discussion

### 4.1. Restriction of Environmental Variables

In this study, nine climate factors and one soil factor were used to predict the suitable areas for *Luprops orientalis* based on the MaxEnt model. The results indicated that bio18, bio04, and bio13 are critical environmental variables influencing its distribution. The findings emphasized the combined importance of the precipitation and temperature variables, which is consistent with previous studies on predicting suitable areas [34,35,36,37]. Furthermore, the response curve analysis revealed that *L. orientalis* exhibited high suitability when the bio18 value ranged from 565.73 mm to 886.55 mm, and the bio13 value ranged from 219.93 mm to 355.67 mm, suggesting a preference for humid environments. The contribution of the land cover type was found to be only 0.8%, suggesting its minimal impact on the distribution pattern of *L. orientalis*. This is consistent with the observed behavior studies, as the species can live both in food storage areas [14,15] and in the wild [19]. Although these 10 environmental variables reflect the optimal conditions for the survival of *L. orientalis*, they do not comprehensively represent the species’ environmental requirements, such as the interactions of unobserved biotic factors or potential abiotic confounding variables [37].

### 4.2. Distribution Pattern of Luprops orientalis Under Near-Current Climate Conditions

Compared with the observed distribution, the predicted suitable areas for *L. orientalis* encompass a significantly larger range, aligning with the findings from previous research [37,38,39,40]. This discrepancy may be attributed to the scope of the survey and the number of distribution points collected. Generally, an increase in the number of distribution points within the target area enhances the accuracy of model predictions [41,42,43]. Under near-current climate conditions, the total suitable area is approximately 3.49 × 10^6^ km^2^. The high-suitability areas (8.22 × 10^5^ km^2^) for *L. orientalis* are consistent with the distribution regions, which are located in North China, Central China, the Korean Peninsula, and Central and Southern Japan. The moderate-suitability areas (1.65 × 10^6^ km^2^) are primarily distributed in Central China, North China, north of the Korean Peninsula, and Japan, and are significantly larger than the low-suitability area (1.02 × 10^6^ km^2^). Based on the near-current suitable habitats, the key regions for managing *L. orientalis* include grain storge areas in North China, Central China, the Korean Peninsula, and Central and Southern Japan.

### 4.3. The Impact of Climate Change on Suitable Habitats of Luprops orientalis

Compared to the near-current suitable area, the prediction indicates an increasing trend in the suitable habitat for *Luprops orientalis* under future climate change, which is consistent with the findings from several previous studies on insects [44,45,46,47]. In the 2050s, as carbon emission concentrations rise, the total suitable habitat area significantly expands, with the increase in low-suitable areas being more pronounced than that in high-suitable areas (Table S2). However, in the 2090s, as carbon emission concentrations continue to escalate, there is a notable decrease in the total suitable habitat area (Table S2). The increase in carbon emissions is expected to diminish the suitable habitat area for various species, aligning with prior studies [48,49,50,51,52].

The suitable habitats for beetles will increase or decrease as the climate warms [53]; however, the suitable habitat area for *Luprops orientalis* will increase in this study. Under future climate change scenarios, the area of high-suitable habitats for *L. orientalis* is projected to decline, while the area of low-suitable habitats increases. This contraction of high-suitable habitats may be closely related to the risks associated with the rising temperature and increased drought in the dry–wet transition zone of Northern China [54]. The northwestern region of Asia is predominantly arid, and although humidity levels in Northwestern China are projected to increase [55], this area remains unsuitable for *L. orientalis*, manifesting only as small pockets of low suitability in the transition area between North China and Northwestern China. The area of moderately suitable habitats remains relatively stable, while high-suitable habitats will continue to decline. The above findings indicate that as climate warming progresses, the suitable habitats for *L. orientalis* are likely to shift towards higher-altitude regions, which corroborates previous conclusions [28,37,40,52,56]. The distribution center is the centroid of all suitable areas, not the region with the most distribution points (such as the Japanese Archipelago and the Korean Peninsula). The number of distribution points is influenced by factors such as the frequency of surveys and the methods of specimen collection.

Consequently, the monitoring and prevention of *L. orientalis* should be strengthened in potential high-suitability areas to mitigate the risk of further invasion and spread. Additionally, *L. orientalis* should be more effectively managed to avoid outbreaks and minimize the economic damage caused to Chinese traditional medicines.

## 5. Conclusions

Utilizing the MaxEnt model, the suitable areas of *Luprops orientalis* were predicted under both near-current and future climate scenarios. Among the 20 environmental variables analyzed, bio18, bio13, and bio4 emerged as significant factors influencing its distribution. Under near-current climate conditions, the suitable areas for *L. orientalis* are mainly distributed in Central China, North China, the Korean Peninsula, and the Japanese archipelago. Under future climate conditions, the total areas of suitable habitats for *L. orientalis* are anticipated to increase, but the high-suitability areas are expected to diminish in the 2050s and 2090s. In the 2050s, while carbon emission concentrations increase, the area of suitable habitats significantly expands. However, in the 2090s, as carbon emission concentrations continue to rise, the area of suitable habitats is projected to decrease. This decline suggests that increased carbon emission concentrations may negatively impact the population size of *L. orientalis*. Additionally, the centroids of the suitable habitats are shifting towards higher latitudes.

## Figures and Tables

**Table 1:** The selected 10 environmental variables used for modeling in this study.

Variable	Variable Description	Contribution (%)
Bio02	Mean daily temperature range	0.2
Bio04	Temperature seasonality	22.2
Bio07	Average annual temperature range	2.3
Bio08	Mean temperature of the wettest quarter	0.8
Bio09	Mean temperature of the driest quarter	2.4
Bio13	Precipitation of the wettest month	9
Bio15	Precipitation seasonality	0.4
Bio18	Precipitation of the warmest quarter	61.3
Bio19	Precipitation of the coldest quarter	0.7
gm-lc-v3	Land cover type	0.8

## Data Availability

The raw data supporting the conclusions of this article will be made available by the authors on request.

## Supplementary Materials

The following supporting information can be downloaded at: https://www.mdpi.com/article/10.3390/insects16060626/s1, Table S1: Potential suitable area of *Luprops orientalis* in China under different scenarios; Table S2: Statistical analysis of the changes in suitable area under different scenarios; Table S3: The latitude and longitude coordinates of the centroids under different scenarios; Figure S1: the ROC curve for predicting the potential distribution range of *Luprops orientalis* based on the MaxEnt model.

## Abbreviations

The following abbreviations are used in this manuscript:

maximum entropy	MaxEnt
area under the curve	AUC
receiver operating characteristic	ROC
