# Supplementary material for: Distribution Pattern and Change Prediction of Luprops orientalis (Coleoptera: Tenebrionidae) Suitable Area in East Asia Under Climate Change
Source: Insects. 2025 Jun 13;16(6):626. doi: 10.3390/insects16060626 (PMC12193915; doi:10.3390/insects16060626)
Supplement: Supplementary file 1 [file insects-16-00626-s001.zip › insects-3611860-supplementary.pdf]

Table S1. Potential suitable area of *Luprops orientalis* in China under different scenarios.

Scenarios, times	Low suitable area (km^2)	Moderate suitable area (km^2)	High suitable area (km^2)
Current	1016408.28	1647166.11	821866.10
SSP126, 2041–2060	1361238.45	1464766.38	626225.68
SSP245, 2041–2060	1315483.83	1594543.67	699358.82
SSP585, 2041–2060	1658658.91	1547706.29	657022.65
SSP126, 2081–2100	1506251.80	1662850.76	733032.85
SSP245, 2081–2100	1597993.05	1396861.46	667757.50
SSP585, 2081–2100	1884740.53	1280464.07	438999.90

Table S2. Statistical analysis of the changes in suitable area under different scenarios.

Scenarios, times	Expansion (%)	Absence in both (%)	Stability (%)	Contraction (%)	Expansion (km^2)	Absence in both (km^2)	Stability (km^2)	Contraction (km^2)
current-2050s, SSP126	1.47	83.29	13.41	1.82	389893.34	22045098.88	3548846.56	482467.72
current-2050s, SSP245	1.55	83.22	14.00	1.23	409719.73	22025272.50	3704742.42	326571.86
current-2050s, SSP585	2.70	82.06	13.82	1.41	715671.12	21719321.11	3657994.16	373320.12
2050s-2090s, SSP126	2.47	82.65	14.21	0.67	652836.33	21874730.27	3761711.27	177028.64
2050s-2090s, SSP245	1.29	83.16	14.39	1.16	341275.15	22010569.21	3808613.23	305848.93
2050s-2090s, SSP585	1.93	81.55	13.21	3.32	509825.06	21582816.17	3495284.58	878380.70

Table S3. The latitude and longitude coordinates of the centroids under different scenarios.

Scenarios, times	Longitude	latitude
current	115.0947888°E	32.99921569°N
20412060-SSP126	115.8504093°E	34.21582584°N
20412060-SSP245	116.0921458°E	34.19452807°N
20412060-SSP585	115.6289156°E	34.80808468°N
20812100-SSP126	115.8108607°E	34.80139715°N
20812100-SSP245	115.8936427°E	34.72899545°N
20812100-SSP585	116.3419409°E	36.24668512°N

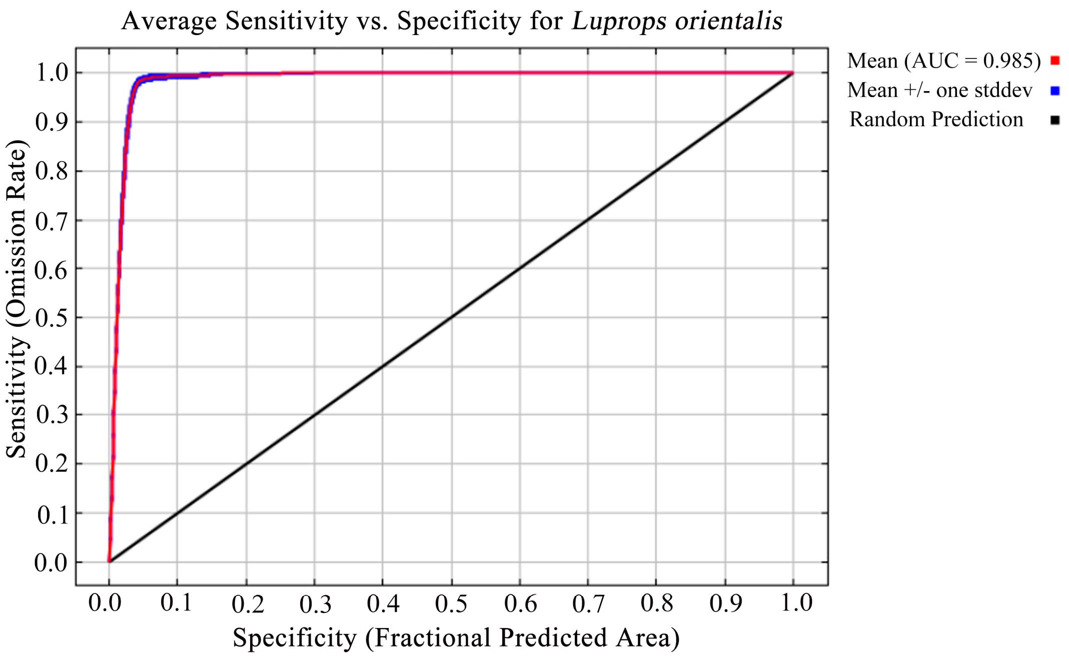

Figure S1. The ROC curve for predicting the potential distribution range of *Luprops orientalis* based on the MaxEnt model.
